# Will calorie restriction work in humans?

**DOI:** 10.18632/aging.100581

**Published:** 2013-07-23

**Authors:** Edda Cava, Luigi Fontana

**Affiliations:** ^1^ Division of Geriatrics and Nutritional Science and Center for Human Nutrition, Washington University School of Medicine, St. Louis, MO 63130, USA; ^2^ Department of Experimental Medicine, Medical Physiopathology and endocrinology Section, Food Science and Human Nutrition Research Unit, University of Rome “Sapienza”, Italy; ^3^ Department of Medicine, University of Salerno Medical School; Biotecnologie Avanzate, Napoli, Italy; ^4^ CEINGE Biotecnologie Avanzate, Napoli, Italy

**Keywords:** calorie restriction, aging, chronic disease, prevention

## Abstract

Calorie Restriction (CR) without malnutrition slows aging and increases average and maximal lifespan in simple model organisms and rodents. In rhesus monkeys long-term CR reduces the incidence of type 2 diabetes, cardiovascular disease and cancer, and protects against age-associated sarcopenia and neurodegeneration. However, so far CR significantly increased average lifespan only in the Wisconsin, but not in the NIA monkey study. Differences in diet composition and study design between the 2 on-going trials may explain the discrepancies in survival and disease. Nevertheless, many of the metabolic and hormonal adaptations that are typical of the long-lived CR rodents did not occur in either the NIA or WNPRC CR monkeys. Whether or not CR will extend lifespan in humans is not yet known, but accumulating data indicate that moderate CR with adequate nutrition has a powerful protective effect against obesity, type 2 diabetes, inflammation, hypertension, cardiovascular disease and reduces metabolic risk factors associated with cancer. Moreover, CR in human beings improves markers of cardiovascular aging, and rejuvenates the skeletal muscle transcriptional profile. More studies are needed to understand the interactions between CR, diet composition, exercise, and other environmental and psychological factors on metabolic and molecular pathways that regulate health and longevity.

Calorie Restriction (CR) without malnutrition is the most powerful nutritional intervention that has consistently been shown to increase maximal and average lifespan in a variety of organisms, including yeasts, worms, flies, spiders, rotifers, fish and rodents [1-7]. Far from merely stretching the life of an old, ill and weak animal, CR extends longevity by preventing chronic diseases, and by preserving metabolic and biological functions at more youthful-like state [6-12]. In rodents, the CR-mediated preventive effects are widespread with major reductions in the occurrence and/or progression of cancer, nephropathy, cardiomyopathy, obesity, type 2 diabetes, neuro-degenerative disease, and several autoimmune diseases [7, 13-19]. Moreover, unlike ad-libitum fed rodents, ~30% of the CR rodents die in old age without any pathological sign of disease [20]. Likewise, 25 to 50% of the longevous Ames/Snell dwarf mice and growth hormone receptor knock-out mice expire without pathological evidence of disease severe enough to be recorded as the cause of death [21-23], suggesting that in mammals the occurrence of lethal chronic disease can be completely prevented by dietary and genetic manipulations that down-regulate the key cellular nutrient-sensing pathways [2]. However, whether or not CR with adequate nutrition will significantly slow aging and extend lifespan in non-human primates, and most importantly in human beings, is not yet clear.

Currently there are two ongoing randomized control studies on the effects of CR without malnutrition in Rhesus monkeys: the Wisconsin National Primate Research Center (WNPRC) and the National Institute on Aging (NIA) CR monkey studies. The WNPRC study, started in 1989, involves 46 male and 30 female rhesus monkeys [24]. The NIA controlled trial, started in 1987, involves 60 male and 60 female rhesus monkeys [25]. Monkeys in both trials have been randomized with a 1:1 ratio to 30% CR or to an ad-libitum diet in the WNPRC and a slight CR diet (to avoid the development of obesity) in the NIA study. Baseline characteristics of the WNPRC and NIA monkeys are recapitulated in Table 1. To date, data from these two trials indicate that long-term 30% CR without malnutrition is feasible and safe in Rhesus monkeys. Importantly, regardless of significant differences in diet composition and study design between the 2 trials, the accumulated data show that CR causes similar metabolic and physiologic adaptations [26], which as we will discuss later emulate only in part those detected in CR mice and rats. Moreover, data on CR-mediated lifespan extension are apparently contradictory in the WNPRC and NIA study.

**Table 1:** Baseline characteristics of the NIA and WNPRC CR monkey studies

	NIA	WNPRC
**Total number of Rhesus monkeys**	120 (60 m, 60 f) 1987: 30 m 1988: 30 m 1992: 60 f	76 (46 m, 30 f) 1989: 30 m 1994: 16 m 1994: 30 f
**Age at baseline**	Juvenile (20 m, 20 f) Adolescent (20 m, 20 f) Old (20 m, 20 f)	All adult
**Animal origin**	India, China	India
**Housing**	Single caged	Single caged
**Randomization**	1 Control: 1 CR	1 Control: 1 CR
**Diet composition and nutrients**	Natural ingredient 3.77 kcal/gr 17,3% P 5% F 56.9% CHO (3.9% from Sucrose) 5-7% crude Fiber Vitamin supplementation +40% of RDA for both CON and CR; all had same diet	Semipurified pellet 3.9 kg/gr 15% P (lactalbumin) 10% F (corn oil) 65% CHO (28.5% from Sucrose) 5% Fiber Vitamin supplementation beyond RDA only for CR monkeys
**Dietary Restriction regimen**	22-24% actual restriction from BL intake levels based on NRC guidelines	30% restriction from a BL intake assessed individually
**Feeding schedule**	Twice a day	Morning ration, plus 100 Kcal integration of food at late afternoon
**Food intake measurement**	1 week per year	Daily quantification for each animal
**Body weight follow-up**	Quarterly under anesthesia; additional measures while awake	Weekly (awake)
**Blood draws**	Every 3 months under anesthesia	Routine blood sampling every 3 months on awake, manually restrained animals. Blood samples are collected under anesthesia only for specific testing (i.e. glucose tolerance testing).

In 2009 the WNPRC group published a paper in Science indicating that long-term (20 yrs) CR reduced by 50% the incidence of cardiovascular disease and cancer, and completely prevented glucose intolerance and type 2 diabetes in rhesus monkeys. They also found that CR protects monkeys against age-associated sarcopenia and grey matter volume shrinkage of several key subcortical regions [27-28]. Similarly, in 2012 Mattison et al. published a paper in Nature indicating that young-onset CR resulted in a complete prevention of cancer, and in a 50% reduction in type 2 diabetes when compared with the mildly restricted control monkeys [29]. In the WNPRC study, CR significantly increased survival when considering only age-associated deaths (i.e. 37% of controls versus 13% of the CR group) [27]. However, when considering all the causes of death, including endometriosis, gastric bloat, complications of anesthesia, and injury, the investigators noted a trend for improved survival that has not reached statistical significance yet (P = 0.16). In contrast, data from the NIA CR monkey study suggest that CR initiated in both young/adult (1-14 yrs) and old (16-23 yrs) animals does not result in a significant improvement in survival [29]. Nonetheless, it should be noted that in the NIA study 4 CR and 1 control monkeys have lived more than 40 years, which is a very long life for a Rhesus monkey [29]. So far 50% of the young-onset monkeys have died, of which 24% of the mildly CR control monkeys have died from age-related diseases compared to 20% of the CR monkeys, and it will take another 10 years before the final data on average and maximal lifespan will be available for both the NIA and WNPRC studies.

The discrepancies in survival and disease incidence between the WNPRC and NIA studies raise several important questions that may help to further understand the mechanisms that regulate aging and mediate health and longevity.

## 1 Is the degree of CR in the NIA and WNPRC monkeys sufficient to trigger an anti-aging effect?

In the WNPRC study, the average difference in body weight between the CR and control monkeys was 3-5 kg in the males, and 1.5-2.5 kg in the females, even if at times the CR monkeys gained a considerable amount of weight and the control monkeys lost weight [30]. In contrast, in the NIA study the difference in body weight between the CR and control monkeys was on average only 2.5 kg [31]. The problem is that in the WNPRC study the CR group calorie intake was individually calculated from the baseline consumption of every monkey, whereas in the NIA study the CR monkeys received an amount of food on the basis of a standard calculation (i.e. recommended caloric intake for age and body weight) for the whole group to accommodate for the growing phase of the young monkeys [26]. In the Wisconsin trial the difference between CR and control group was kept around 30% of food intake for the males and around 25% for the females, adjusting the restricted-diet intake whether there was a period of lowered intake among the control group. However, as the monkeys got older the practice of group-wide reductions in the CR animal allotments when controls voluntarily reduced their intakes, has been discontinued, and food intake adjustments for the CR group have been based on animal health. In the NIA study, on the other hand, the actual food intake of the CR group had been reduced only 22-24% below control levels and this the mild restriction of the control group is based on the fact that the monkeys received a controlled allotment of food each day, they were not freely fed with access to food all day [26].

Interestingly, in both the NIA and WNPRC studies many of the metabolic and hormonal adaptations that are typical of the long-lived CR mice and rats did not occur in the CR monkeys. In the NIA study, unlike in CR rodents, serum concentrations of glucose, total cholesterol, LDL-cholesterol, C-reactive protein, testosterone and estrogen were not reduced, and serum cortisol were not increased in the CR monkeys [29, 32-33]. Similarly, no difference in serum concentrations of total cholesterol, LDL-cholesterol, HDL-cholesterol, triiodothyronine, DHEA, IGF-1, or cortisol have been found between the CR and control monkeys in the WNPRC study [34]. No data have been published yet on the effects of CR on serum testosterone and estradiol concentration in the WNPRC study. How can we explain the difference in metabolic and hormonal adaptations in response to CR between CR rodents and monkeys? Can this discrepancy be explained by species differences? The data collected in lean humans practicing long-term (on average 7 years) CR without malnutrition argue against this hypothesis, and suggest that the degree of CR in the two Rhesus monkey studies might be insufficient to trigger the classical CR-mediated metabolic/hormonal reprogramming that mediate longevity in rodents. In fact, we found that serum concentrations of glucose, total cholesterol, LDL-cholesterol, triiodothyronine, testosterone and estradiol were significantly lower in lean individuals practicing long-term moderate CR (i.e. CRONies) than in age- and sex-matched controls [35-37]. In contrast, the NIA-funded “CALERIE phase 1” randomized clinical trials have failed to demonstrate a significant reduction in serum IGF-1, testosterone and estradiol, and an increase in cortisol levels in men and women who underwent 20% CR for 6-12 months [38-41]. The truth is that the CALERIE phase 1 trials were just weight loss studies in overweight men and women who lost some body weight and body fat (average BMI and body fat at the end of the study was ~24 kg/m^2^ and 29%, respectively), but not enough to elicit the distinctive metabolic, hormonal and molecular adaptations induced by moderate/severe CR in lean rodents and in the CRONies (average BMI and body fat was 19.6 kg/m^2^ and 12%, respectively). It is our working hypothesis that in order to trigger a powerful anti-aging response, the body needs to perceive a CR-induced low energy availability that results in major simultaneous metabolic adaptations (i.e. low circulating levels of leptin, insulin, IGF-1, testosterone, estradiol, triiodothyronine and inflammatory cytokines coupled with increased serum concentrations of adiponectin, ghrelin and cortisol) with energy resources shifted from growth and reproduction towards maintenance and repair activities. Interestingly, leanness (low adiposity) induced by chronic exercise training (i.e. high energy expenditure coupled with high energy intake) also does not result in some of the same key metabolic adaptations that have been hypothesized to play a role in the CR-induced longevity, including a decrease in triiodothyronine, IGF-1 and core body temperature [36, 38, 42]. Consistently, data from experimental murine studies have shown that only CR slows aging and extends both average and maximal lifespan, whereas life-long exercise extends only average lifespan [43].

## 2 Is diet composition as important as calorie intake in mediating healthy longevity?

A remarkable difference between the NIA and WNPRC trials has been diet composition. The Wisconsin monkeys ate a pellet semi purified diet providing 3.9 kcal/g, with 15% calorie from dairy proteins (i.e lactalbumin), 10% calories from oil (i.e. corn oil), and approximately 65% calories from refined and processed carbohydrates (principally sucrose and cornstarch)[24]. In contrast, the NIA monkey diets are based on natural ingredients calculated on estimated requirements for nonhuman primates, with soy oil, fish, wheat, corn and alfalfa meal as fat source. A mixture of fish, soybean, wheat, corn, and alfalfa meal provided the great majority of protein in the NIA study, and carbohydrates were primarily from ground wheat and corn [44]. Sucrose was only 3.9% in the NIA study, while the WNPRC diet was 28.5% sucrose. The NIA diet macronutrient composition was 56.8% carbohydrates, 17.3% protein, 5% fat, and 5-7% crude fiber, with an energy density of 3.77 kcal/g. Concentration of vitamins and minerals were increased by 40% above the adequate amount for both the CR and control animals, to be sure to meet the 100 percent of the recommended daily allowance and eliminate variables between the groups. In contrast, in the WNPRC study only the CR monkey diets were supplemented. Moreover, in contrast to the WNPRC semi-purified diets, the NIA natural ingredient-based diets contain a wide variety of phytochemicals, minerals, and omega-3 fatty acids, which are known to have independent positive health effects on several metabolic pathways [45-47]. For examples, certain plant foods contain a wide range of phytochemicals (i.e. polyphenols, catechins, stilbenes, isothiocyanates, terpenes, sterols, indoles, and organosulfur compounds) and vitamins that have shown beneficial effects against inflammation, oxidative stress, and on other molecular pathways that regulate blood pressure, atherosclerosis and carcinogenesis [48]. In summary, the WNPRC diet resembles more closely the typical modern Western diet rich in refined and processed foods; in contrast, the NIA diet is more similar to the traditional Mediterranean or Japanese diet, rich in fish and minimally processed plant-based foods. Therefore, it is possible that the beneficial effects on lifespan of the combination of phytochemical-rich pescovegetarian diets and mild CR in the NIA control monkeys are already maximized. Consistently, average lifespan for both the NIA CR and control monkeys was markedly higher (35.4 years for males and 27.8 years for females) than in Rhesus monkeys in captivity (~27 years), with endometriosis (a nonlethal disease in humans) being the biggest factor leading to a shorter lifespan in females than males [29].

## 3 Is protein intake a key determinant of longevity in CR primates?

Accumulating data suggest that protein intake and dietary aminoacid composition play an important role in regulating mTOR activity, serum IGF-1 concentrations, and longevity [2, 38, 49-54]. Our data show that in humans, unlike in rodents, severe CR does not reduce serum IGF-1 concentration unless protein intake is also reduced close to the USDA recommended intake (i.e. 10% calories from protein, or 0.8 g/kg/day) [38]. Data from genetic animal and human studies indicate that serum IGF-1 concentration is an important regulator of aging [2, 55-56], and has been found to be inversely correlated with median lifespan in 31 genetically diverse inbred mouse strains [57]. Interestingly, back in 1950 the Okinawan centenarians were consuming a CR diet (approximately 1800 cal/day) with only 9% of calories coming from protein [58]. In contrast, both the WNPRC and NIA monkey diets contain 15% or more calorie from protein, which is similar to the average protein intake of US men and women [59], and may explain why serum IGF-1 concentrations were similar between CR and control monkeys. More studies are needed to understand the role of protein intake (and aminoacid composition of foods) with and without CR in regulating the pro-longevity PI3K/AKT and mTOR pathways in rodents and primates. The old dogma that only calorie intake, and not macronutrient composition (and in particular protein intake), is an important regulator of lifespan is based on a flawed interpretation of a study published by the Masoro's group in 1985. In this experiment the authors restricted calorie intake in one group of rats by 40% and compared them to rats given free access to a diet in which protein content was reduced by 40%. The control group ate a usual protein content diet ad libitum [60]. In this experiment the 40% CR diet increased maximal longevity ~35%, while the reduced protein diet had no effect. The control diet provided 20% of calories from protein, compared to 12% of calories from protein for the “protein restricted” diet. The problem the authors failed to recognize is that, despite the 40% reduction, protein intake was still more than adequate, i.e. was above the threshold needed to cause an inhibition of the IGF/mTOR pathway, as evidenced by the finding that weight gain for these young, growing animals was the same in the 12% and the 20% protein diet groups [60]. In contrast, the CR animals gained little weight and were markedly stunted as adults, suggesting that only 40% CR and not 40% protein restriction, inhibits the PI3K/AKT/mTOR pathways.

Despite Rhesus monkeys (Macaca mulatta) representing one of the most closely related species to human primates, sharing a ~93% DNA sequence identity with the human genome [61], major differences in longevity exist between monkeys and humans. Even under the best husbandry and dietary conditions (i.e NIA CR monkey study), average and maximal lifespan of rhesus monkeys is ~31 and ~40 years, respectively. In contrast, average and maximal lifespan in humans is ~80 and ~120 years, respectively. The reason why Rhesus monkeys lifespan is much shorter than in humans is not known, and may involve a different rate of accumulation of unrepaired molecular and cellular damage with time. Therefore, it is extremely important to study the health and longevity effects of CR without malnutrition in humans.

Whether or not CR without malnutrition will extend lifespan in humans is not known yet, but accumulating data indicate that moderate CR with adequate nutrition has a powerful protective effect against the development of obesity, type 2 diabetes, inflammation, hypertension and cardiovascular disease, which are major causes of morbidity, disability and mortality [37]. Accordingly, Lloyd-Jones and colleagues found that in men and women from the Framingham Heart Study with normal cardiovascular risk profile at age 50 (i.e. total glycemia <125 mg/dl, blood pressure <120/80 mmHg, cholesterol <180 mg/dl, BMI <25 kg/m^2^ and no smoke) the lifetime probability of developing an atherosclerotic cardiovascular disease was very low (i.e., 6.7% versus 59.5% in participants with ≥2 cardiometabolic risk factors) and average lifespan markedly longer (i.e. >39 versus 29.5 years in participants with ≥2 cardiometabolic risk factors) [62]. In humans calorie restriction without malnutrition also results in a consistent reduction in circulating levels of growth factors, anabolic hormones, adipokines and inflammatory cytokines, which are associated with an increased risk of some of the most common types of cancer [63]. It is important to note that none of the 50 men and women (age range 30-82 yrs) practicing long-term CR with adequate nutrition is taking any medication or has developed any chronic disease so far. Moreover, CR in these individuals resulted in an amelioration of two well-accepted markers of cardiovascular aging, i.e. left ventricular diastolic function and heart rate variability [64-65]. These data indicate that CR exerts direct systemic effects that counter the expected age-associated changes in myocardial stiffness and autonomic function so that LV diastolic function and heart rate variability indexes in CR individuals are similar to those of individuals 20 years younger on a typical Western diet. Consistently, we recently found that CR without malnutrition results in dramatic changes of the human skeletal muscle transcriptional profile that resemble those of younger individuals, including a down-regulation of the PI3K/Akt/FOXO pathway, suggesting that CR in humans can slow the age-associated transcriptional modifications in skeletal muscle [66].

More studies are needed to understand how macro- and micro-nutrients, endurance exercise, and other environmental and psychological factors interact with CR in modulating metabolic and molecular pathways that regulate health and longevity. Both excessive dietary restriction and overnutrition are different forms of malnutrition that lead to organ dysfunction and increased mortality. Even in rodents, excessive CR imposed on some strains of mice increases mortality. For example, it has been shown that wild-caught mice undergoing 40% CR do not live longer than ad-libitum fed mice, despite a much lower cancer incidence [67]. This may be caused by excessive CR during the developmental age, because 40% CR caused higher mortality early in life, but lower mortality late in life, with the longest lived 8% of mice all coming from the CR wild-caught rodents. Furthermore, in C57BL/6J mice 40% CR increases mortality when started just after weaning (i.e. 4 weeks of age), but increase lifespan when started in middle age [68-69]. The problem is that the rate of physiologic development and sexual maturation varies among different strains of rodents, so that the lifespan response to CR may be different. Forty percent CR may be optimal in some strains of mice, but can cause severe starvation and increased mortality in others, which would benefit from a lower degree of CR. The same applies to humans, in which severe CR could be detrimental in some populations (e.g. children, older adults, pregnant women, etc.). Additional studies are warranted to identify the precise CR-induced metabolic and molecular adaptations associated with healthy longevity, so that dietary energy content and macro-nutrient composition can be tailored based on age, sex, disease predisposition and biological/genetic phenotype of each individual.

Randomized, CR-controlled, long-term survival studies in humans will never be performed because of obvious problems with long-term compliance and costs of such a long study. Nonetheless, we hope that by following the health status of individuals practicing long-term CR without malnutrition (i.e. the CRONies), in particular of those who are now in their 70s and 80s, we could gain soon some information about the effects of CR on successful aging and healthy longevity in humans as well. Because we have detailed information about their close relatives' disease and survival histories, if we observe that as the CRONies age, they not develop any of the metabolic abnormalities and/or chronic diseases typical of their parents/siblings, and live substantially longer than their relatives, this will be the best available proof that CR works in humans.
